# Impaired inhibition of return during free-viewing behaviour in patients with schizophrenia

**DOI:** 10.1038/s41598-021-82253-w

**Published:** 2021-02-05

**Authors:** Ken-ichi Okada, Kenichiro Miura, Michiko Fujimoto, Kentaro Morita, Masatoshi Yoshida, Hidenaga Yamamori, Yuka Yasuda, Masao Iwase, Mikio Inagaki, Takashi Shinozaki, Ichiro Fujita, Ryota Hashimoto

**Affiliations:** 1 Graduate School of Frontier Biosciences, Osaka University, Osaka, 565-0871 Japan; 2 Center for Information and Neural Networks (CiNet), National Institute of Information and Communications Technology, and Osaka University, Osaka, 565-0871 Japan; 3 Department of Pathology of Mental Diseases, National Institute of Mental Health, National Center of Neurology and Psychiatry, Ogawa-Higashi 4-1-1, Kodaira, Tokyo, 187-8553 Japan; 4 Department of Psychiatry, Osaka University Graduate School of Medicine, Osaka, 565-0871 Japan; 5 Department of Rehabilitation, University of Tokyo Hospital, Tokyo, 113-8655 Japan; 6 Department of Developmental Physiology, National Institute for Physiological Sciences, Aichi, 444-8585 Japan; 7 School of Life Science, The Graduate University for Advanced Studies, Kanagawa, 240-0193 Japan; 8 Center for Human Nature, Artificial Intelligence, and Neuroscience, Hokkaido University, Hokkaido, 060-0812 Japan; 9 Japan Community Health Care Organization Osaka Hospital, Osaka, 553-0003 Japan; 10 Life Grow Brilliant Mental Clinic, Medical Corporation Foster, Osaka, 530-0012 Japan; 11 Molecular Research Center for Children’s Mental Development, United Graduate School of Child Development, Osaka University, Osaka, 565-0871 Japan; 12 Graduate School of Information Science and Technology, Osaka University, Osaka, 565-0871 Japan; 13 Present Address: Department of Physiology, Hokkaido University School of Medicine, Hokkaido, 060-8638 Japan

**Keywords:** Diseases of the nervous system, Schizophrenia, Neuroscience, Oculomotor system, Saccades, Diseases, Psychiatric disorders, Schizophrenia, Biomarkers, Medical research, Biomarkers

## Abstract

Schizophrenia affects various aspects of cognitive and behavioural functioning. Eye movement abnormalities are commonly observed in patients with schizophrenia (SZs). Here we examined whether such abnormalities reflect an anomaly in inhibition of return (IOR), the mechanism that inhibits orienting to previously fixated or attended locations. We analyzed spatiotemporal patterns of eye movement during free-viewing of visual images including natural scenes, geometrical patterns, and pseudorandom noise in SZs and healthy control participants (HCs). SZs made saccades to previously fixated locations more frequently than HCs. The time lapse from the preceding saccade was longer for return saccades than for forward saccades in both SZs and HCs, but the difference was smaller in SZs. SZs explored a smaller area than HCs. Generalized linear mixed-effect model analysis indicated that the frequent return saccades served to confine SZs’ visual exploration to localized regions. The higher probability of return saccades in SZs was related to cognitive decline after disease onset but not to the dose of prescribed antipsychotics. We conclude that SZs exhibited attenuated IOR under free-viewing conditions, which led to restricted scene scanning. IOR attenuation will be a useful clue for detecting impairment in attention/orienting control and accompanying cognitive decline in schizophrenia.

## Introduction

Patients with schizophrenia (SZs) commonly show deficits in eye movement control that manifest as atypical visual exploration^1–4^. Eye movement abnormalities in SZs have been proposed as a biomarker reflecting the underlying pathophysiology of the disorder^5–8^. For example, difficulties exhibited by SZs in performing smooth pursuit are related to various cognitive dysfunctions in visual motion processing and predictive control^9^, while problems with antisaccade are associated with working memory and inhibitory control^10,11^. Atypical visual exploration in SZs reportedly correlates with disturbed performance in a number of cognitive tests that depend on the proper gathering and processing of visual information. These tests include facial expression recognition^12^, the Wechsler Adult Intelligence Scale picture completion test^13^, the Benton Visual Retention test^14^, and matrix reasoning tasks^15^. Possible psychopathological deficits underlying atypical visual exploration in SZs include aberrant computation of salience of visual scenes^16^; reduced motivation, which is a characteristic of negative symptoms^17^; and cognitive deficits in working memory and/or selective attention^18^. Visual exploration consists of saccades, which shift the gaze via quick eye movements, and fixations, which maintain the gaze on a single location. SZs’ atypical visual exploration is characterized by lower saccade frequency, smaller saccade amplitude, and longer fixation duration^6,19–22^. It remains unclear whether these features alone can sufficiently explain the atypical visual exploration in SZs, or whether there is another feature reflecting the deficit in their attention/orienting system. One such possible feature is repeated visits to previously explored regions, possibly reflecting an abnormality in inhibition of return (IOR).

IOR is a mechanism that inhibits orientation to previously fixated or attended locations in the visual scene^23^. In the classical Posner’s paradigm, an uninformative cue is presented to either the same or another location prior to a target stimulus^24^. The reaction time to the target is delayed in the same-location trials (i.e., reflecting IOR) when the interval between the cue and target is longer than 300 ms. Later studies extended the concept of IOR to natural visual exploration by showing that saccades towards previously fixated locations (return saccades) occurred slowly and less frequently in visual search^23,25^. Klein and colleagues proposed the ‘foraging facilitator’ hypothesis, which posits that IOR limits repeated visits to previously explored sites by inhibiting saliency of the items in multiple previously fixated locations, and facilitates foraging of new visual information, thus enabling the efficient search of visual scenes^23,25^. Subsequent studies reported that return saccades were less frequent than would be estimated by chance^26,27^, but more frequent than saccades in orthogonal directions^28,29^. Current models of visual search incorporate IOR mechanisms to reproduce efficient and human-like performance^30,31^. In these models, the local salience of a scene is a critical determinant of the location of coming saccades, and IOR is implemented to suppress saccades toward previously fixated locations.

SZs exhibit weak and delayed IOR in the Posner’s paradigm^32–34^. However, no studies thus far have examined IOR in SZs during natural visual exploration. Here we studied whether SZs showed impaired IOR in this context, and if so, whether their exploration of a scene was affected. We examined IOR in SZs and healthy control participants (HCs) by analysing the spatial and temporal properties of saccades during free viewing of visual images. We further analysed the contribution of IOR to efficient visual search by constructing generalized linear mixed-effect models (GLMEs), and assessed the relationship between IOR and SZs’ demographic and clinical characteristics by constructing generalized linear models (GLMs).

## Methods

### Participants and ethics

Data were sampled from 122 SZs (male, 60; female, 62) and 490 HCs (male, 265; female, 225) as part of a large-scale cohort recruited at Osaka University (Table 1)^6,15,22^. The individuals were not related, all were of Japanese descent, and none had a history of ophthalmologic or other neurological diseases. Specific exclusion criteria included atypical headaches, head trauma with loss of consciousness, chronic lung disease, kidney disease, chronic hepatic disease, thyroid disease, active cancer, cerebrovascular disease, epilepsy, seizures, substance-related disorders, or mental retardation. SZs had been diagnosed by two or more trained psychiatrists according to the criteria of the Diagnostic and Statistical Manual of Mental Disorders, 4th Edition (DSM-IV)^35^ based on the Structured Clinical Interview for DSM-IV.

**Table 1:** Demographic and clinical characteristics of HCs and SZs.

	Healthy controls (N = 490)	Schizophrenia patients (N = 122)	*p* value*	d′
	Mean ± SD	Mean ± SD		
**Demographics**				
Age (years)	32.0 ± 14.5	35.6 ± 12.1	0.01	0.26
Sex (male/female)	265/225	60/62	0.33**	
Education (years)	14.8 ± 1.8	14.0 ± 2.6	< 0.001	0.41
Current IQ	113.3 ± 12.1	88.7 ± 15.9	< 0.001	1.91
Estimated premorbid IQ		102.6 ± 9.2		
Cognitive decline		− 13.9 ± 12.4		
Onset age (years)		22.9 ± 10.4		
Duration of illness (years)		12.6 ± 9.9		
**Symptoms**				
PANSS total		83.5 ± 20.2		
PANSS positive		19.1 ± 5.7		
PANSS negative		20.9 ± 5.1		
PANSS general		43.5 ± 10.6		
**Drug (mg/day)**				
CPZ eq.		531.4 ± 513.7		
Typical antipsychotic eq.		158.4 ± 363.3		
Atypical antipsychotic eq.		376.0 ± 489.1		
Diazepam eq.		7.9 ± 12.0		
Biperiden eq.		0.8 ± 1.2		

*Based on *t*-test except for Sex, **χ^2^-test.

*PANSS* Positive and Negative Syndrome Scale, *CPZ eq*. chlorpromazine equivalents.

The symptoms of SZs were evaluated using the Positive and Negative Syndrome Scale (PANSS)^36^. The total amount of prescribed antipsychotics was calculated using chlorpromazine (CPZ) equivalents (mg/day). Psychotropic dose equivalencies for typical antipsychotics, atypical antipsychotics, diazepam, and biperiden were also determined^37^. IQ was measured using the Japanese version of the Wechsler Adult Intelligence Scale-Third Edition^38^. The premorbid IQ of SZs was estimated using the Japanese version of the National Adult Reading Test^39^. The rationale behind using this tool is that reading ability is relatively intact in SZs^40^. The difference between premorbid and current IQ provided an estimate of cognitive decline in the SZs^41,42^, although this indirect measure may not fully probe the whole range of cognitive deficits^42^.

This study was performed in accordance with the Declaration of Helsinki and was approved by the Research Ethical Committee of Osaka University. Informed consent was obtained from all participants to participate in the study after full explanation of the study procedures. Anonymity was preserved for all participants.

### Behavioural paradigm

The participants faced a 19-inch liquid crystal display placed at a distance of 70 cm from their eyes. The display covered a visual angle of 32° × 24°. Visual stimuli were presented using MATLAB (Mathworks, Natick, MA, USA) via the Psychophysics Toolbox extension^43^.

The participants freely viewed 56 images, one at a time for 8 s, from eight categories, including natural environments, animals, faces, buildings, everyday items, foods, geometrical patterns, and pink noise. Each category consisted of seven images. Images of natural environments and animals were selected from the International Affective Pictures System^44^. Images of faces were selected from the study of Matsumoto and Ekman^45^. The pink-noise images, in which some spatial structure was apparent, consisted of pseudo-random noise with a 1/f power spectrum density similar to that of the natural images.

A trial started when a participant fixated a central white point on the gray background to a precision of ± 3°. One second after the fixation point disappeared, one of the 56 images was presented in random order across trials. The participants were instructed to freely view the image for a period of 8 s. Each image was presented only once. About 10 min were required to complete the entire task.

### Eye movement data processing

**Figure 1:**
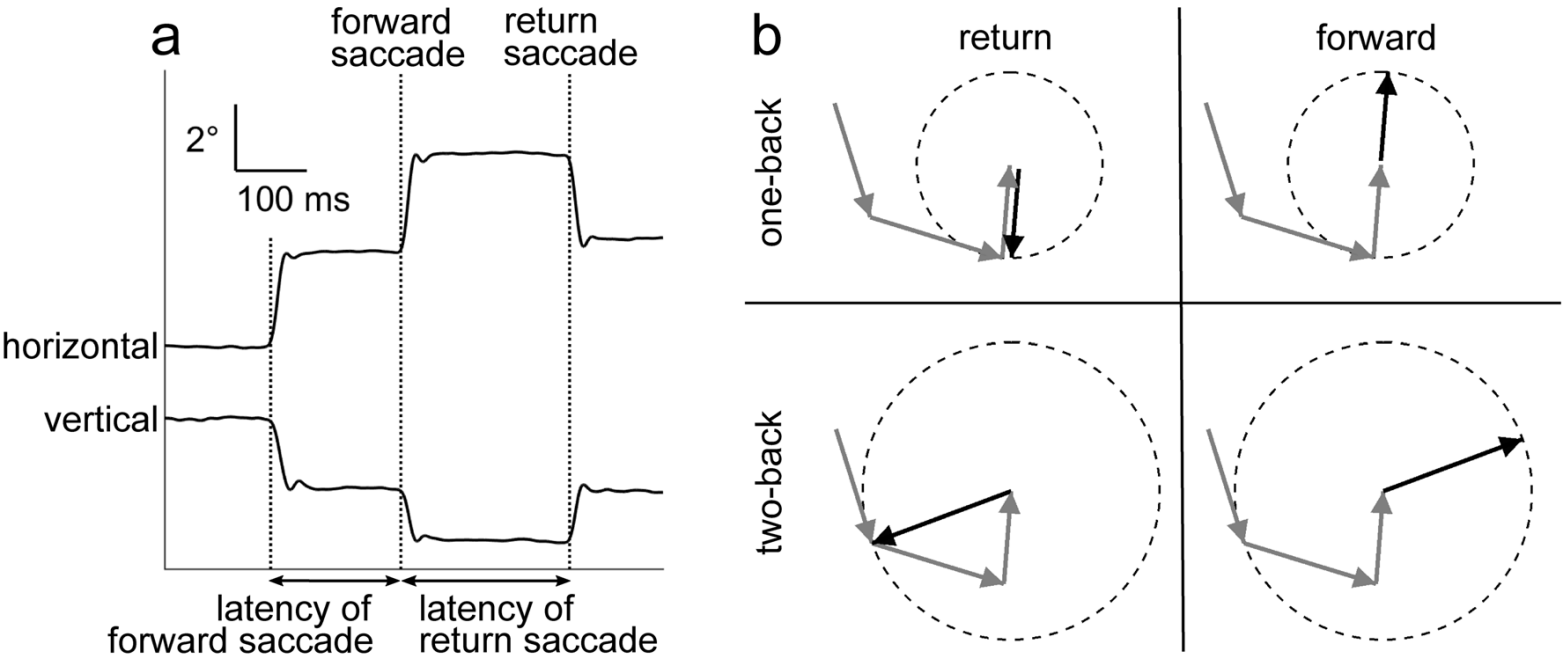
(**a**) Representative eye trace of an SZ during free-viewing behaviour. Horizontal and vertical eye positions are shown. We defined a return saccade as movement of the eyes in the opposite direction of the previous saccade to the originally fixated location. A forward saccade was defined as movement of the eyes in the same direction and with the same amplitude. We defined the latency of a saccade as the time lapse from the previous saccade, i.e., the duration of the preceding fixation. (**b**) Schematic illustration of return and forward saccades. Gray arrows indicate three sequential preceding saccades. Black solid arrows represent return (left) and forward (right) saccades relative to one-back (top) and two-back (bottom) fixation locations, respectively. Dashed circles represent a relative amplitude of 1.

Eye movements and the pupil area of the left eye were measured at a sampling rate of 1 kHz using the EyeLink 1000 or 1000-plus system (SR Research, Ottawa, Ontario, Canada). Eye position data were smoothed with a digital finite impulse response filter (− 3 dB at 30 Hz). Saccade periods were determined based on the high-speed changes of eye position (velocity > 35°/s; acceleration > 5000°/s^2^). To examine the effect of previous fixations on subsequent behaviour, we used the total of 448 s of eye trajectory data (8 s × 56 images) to extract the amplitude and direction of saccades relative to the previous fixation locations. A return saccade directed to the one-back fixation location was defined as a saccade in the opposite direction of the previous saccade (180° ± 30°) with a similar amplitude (amplitude difference < 1°) (Fig. 1). We selected this criterion based on the mean saccade distribution in HCs and SZs (see Fig. 3). Notably, when we relaxed the criterion to an amplitude difference of < 2°, we obtained qualitatively identical results. A forward saccade was defined as eye movement in the same direction as the previous saccade (direction difference < 30°) with a similar amplitude (amplitude difference < 1°). Saccade latency was defined as the time elapsed from the preceding saccade (Fig. 1a). We evaluated impairment of IOR in SZs by examining the probability and latency of return saccades. Return saccades directed to two-back or three-back fixation locations were also investigated (Fig. 1b). Any data affected by eye blinks were omitted from analyses.

### Statistical analysis

Comparisons between SZs and HCs in terms of return saccade probability and other eye movement parameters were made using the non-parametric Mann–Whitney *U*-test. The diagnostic ability of each parameter was estimated by the effect size determined by Cohen’s *d*^46^ and the area under the curve (AUC) calculated by receiver operating characteristic (ROC) analysis^47^. For individual participants, comparison of the number of return and forward saccades was performed by the *F*-test for two counts. To examine the effect of relative saccade direction on the probability of similar amplitude saccades, the Friedman test was performed for analysis of nonparametric repeated variables, and the repeated Wilcoxon signed-rank test after Bonferroni correction was performed for post-hoc analysis.

SZs exhibit a deficit in computing salience in an image^16^. It is possible that SZs may exhibit frequent return saccades as a result of overestimating image salience and failing to suppress return saccades to the overestimated image sites. To determine whether image salience alone could explain the difference in return saccade frequency between SZs and HCs, we analysed eye movement patterns when the participants viewed 1/f noise images that had only a weak fluctuation of salience across space. We computed the saliency map for each noise image using the *MATLAB saliency toolbox*^30,48^, and compared the salience of the fixated locations between SZs and HCs and between return and no-return saccade locations by two-way analyses of variance (ANOVA).

To evaluate the magnitude of the delay of return saccades, we calculated the return latency index as the mean latency of return saccades *z*-scored by the mean latency of forward saccades.

The efficiency of visual search of an explored area during an 8-s trial was determined by calculating the proportion (%) of the area enclosed by the outermost fixation locations relative to the entire image area (Fig. 5a,b). We compared the median explored area over 56 images for HCs and SZs.

### Generalized linear model analysis

Because the return saccade probability across subjects was not normally distributed, simple correlation coefficients could not be used to examine the relationship between the return saccade probability and either the return latency index or the demographic/clinical variables. Thus, we analysed the relationship between the return saccade probability and other eye movement parameters and demographic data by constructing a GLM or GLME^49^ with a gamma distribution and a log link function using the *fitglm* or *fitglme* function in MATLAB. In the analysis in Fig. 4b, we modeled the log-transformed return saccade probability as a linear function of intercept, return latency index, participant group (HCs or SZs), and interaction between return latency index and participant group, as follows.

$$log\;(Return\; saccade \;probability) \sim Return\; latency\; index + Group + Interaction.$$

After we found that SZs exhibited impaired IOR in free-viewing conditions, we addressed whether this deficit reduced the efficiency of SZs’ visual searches, as assumed by the foraging facilitator hypothesis^23,25^. To examine the relationship between exploration area and saccade parameters, we used GLME to regress the exploration area for each image category on saccade parameters to account for the repeated observations of the same subjects. We constructed a GLME model to estimate how the saccade amplitude (SacAmp), saccade number (SacNum), return saccade probability (RSac), and participant group (Group) contributed to the exploration area in each image category, as follows.

$$log\;(Area) \sim SacAmp + SacNum + RSac + Group$$

SacAmp, SacNum, and RSac were *z*-scored before application to the model. We accounted for individual differences in exploration area across subjects and image categories as random effects. The relationships between SZs’ return saccade probability and their demographic and clinical characteristics were determined by constructing GLMs separately for HCs and SZs.

## Results

The eye movement trajectories of 122 SZs and 490 HCs were examined during free viewing of natural and artificial images. The demographic and clinical data are summarised in Table 1. SZs showed a smaller saccade amplitude (mean ± s.d., 3.20° ± 1.10° for SZs, 3.57° ± 1.00° for HCs; Mann–Whitney *U*-test, *z* = 3.61, *p* < 0.001, *d’* = 0.36, AUC = 0.61 by ROC analysis), smaller saccade number (1.82 ± 0.46/s for SZs, 2.24 ± 0.44/s for HCs; *z* = 8.54, *p* < 0.001, *d’* = 0.96, AUC = 0.75), and longer duration of fixation (290 ± 47 ms for SZs, 255 ± 36 ms for HCs; *z* = 7.93, *p* < 0.001, *d’* = 0.90, AUC = 0.73) compared to HCs.

### SZs show more frequent and less-delayed return saccades than HCs in free-viewing conditions

**Figure 2:**
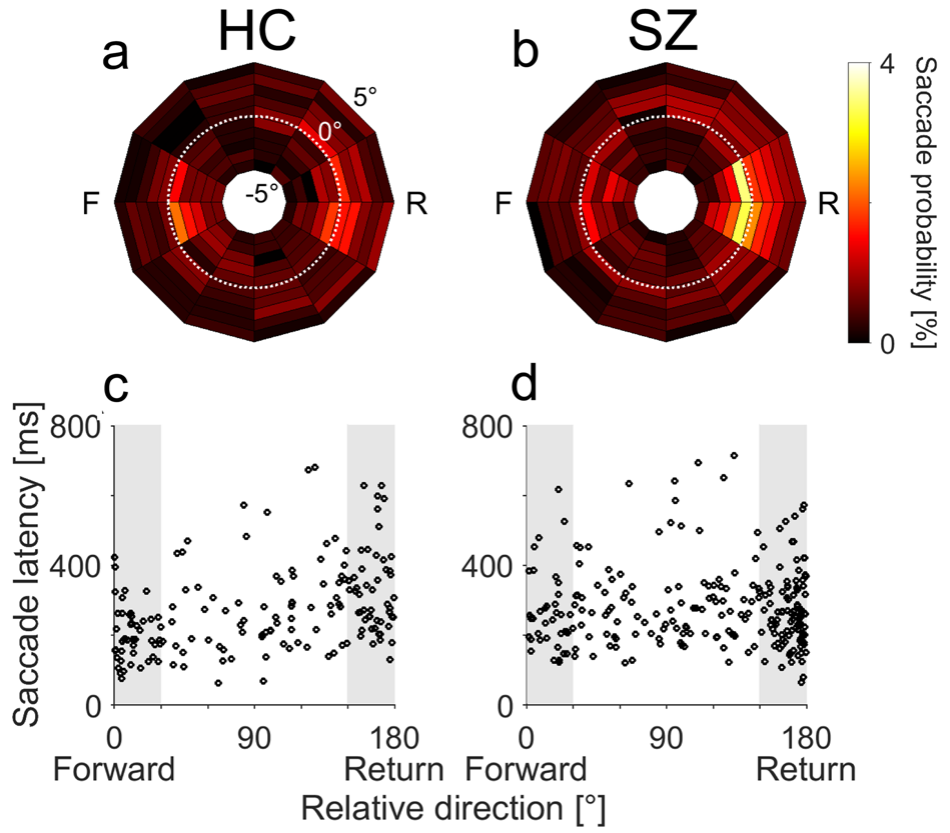
(**a**,**b**) The distributions of the amplitudes and directions of individual saccades relative to their respective one-back saccades for a representative HC (**a**) and SZ (**b**). Probabilities of saccades with different directions (denoted by the angles of the polar plots; 30° bins) and amplitudes (denoted by the radius; 1° bins, range − 5° to 5°) relative to the preceding saccades are shown as heatmaps. White dotted lines represent an amplitude difference of 0°, indicating that a saccade has the same amplitude as the preceding saccade. F and R indicate forward and return directions, respectively. (**a**) The HC made both return and forward saccades more frequently than saccades in other directions. (**b**) The SZ exhibited more return saccades than forward saccades. (**c**,**d**) The saccade latencies are plotted against the relative saccade directions for amplitude-matched (< 1°) sequential saccades for the same HC (**c**) and SZ (**d**). Data within the shaded areas are defined as forward (left) and return (right) saccades.

SZs showed signs of weaker IOR in their visual exploration patterns. Figure 2 illustrates characteristic differences between an HC and an SZ in terms of the distribution of individual saccades relative to the previous fixation location. The HC made both return and forward saccades more frequently than saccades in other directions, and there was no statistically significant difference between the occurrences of return and forward saccades (Fig. 2a, 52 and 45 for return and forward saccades, respectively; *F*-test for two counts, *F* = 1.13, *p* = 1.00). By contrast, the SZ (Fig. 2b) exhibited more frequent return saccades than forward saccades (103 and 39 for return and forward saccades, respectively; *F* = 2.58, *p* < 0.001). Forty-three percent of SZs (52/122) exhibited significantly more frequent return saccades than forward saccades, indicating weak IOR (Supplementary Table S1). This proportion was larger than that for HCs (126/490, 26%; χ^2^-test, χ^2^ = 13.5, *p* < 0.001).

For the HC, the latencies (time lapses from the preceding saccades) of return saccades were longer than those of forward saccades (Fig. 2c; mean ± s.d., 346 ± 129 ms and 271 ± 165 ms for return and forward saccades, respectively; Mann–Whitney *U*-test, *z* = 3.00, *p* = 0.003). For the SZ, there was no difference between the return and forward saccade latencies (Fig. 2d; 268 ± 110 ms and 275 ± 108 ms for return and forward saccades, respectively; *z* = 0.32, *p* = 0.75). The proportion of participants who exhibited significantly longer latencies for return saccades than for forward saccades, which is another indication of strong IOR, was smaller in SZs (70/122, 57%) than in HCs (361/490, 74%; *χ*^2^-test, χ^2^ = 12.5, *p* < 0.001, Supplementary Table S1).

Overall, the proportion of participants who exhibited strong IOR as manifested by equal or lower return saccade probability and by delayed return saccade latency was smaller in SZs (52/122, 42%) than in HCs (290/490, 59%; *χ*^2^-test, χ^2^ = 10.9, *p* < 0.001). In other words, the remaining 58% of SZs showed attenuated IOR as manifested by higher return saccade probability, less-delayed return saccade latency, or both.

**Figure 3:**
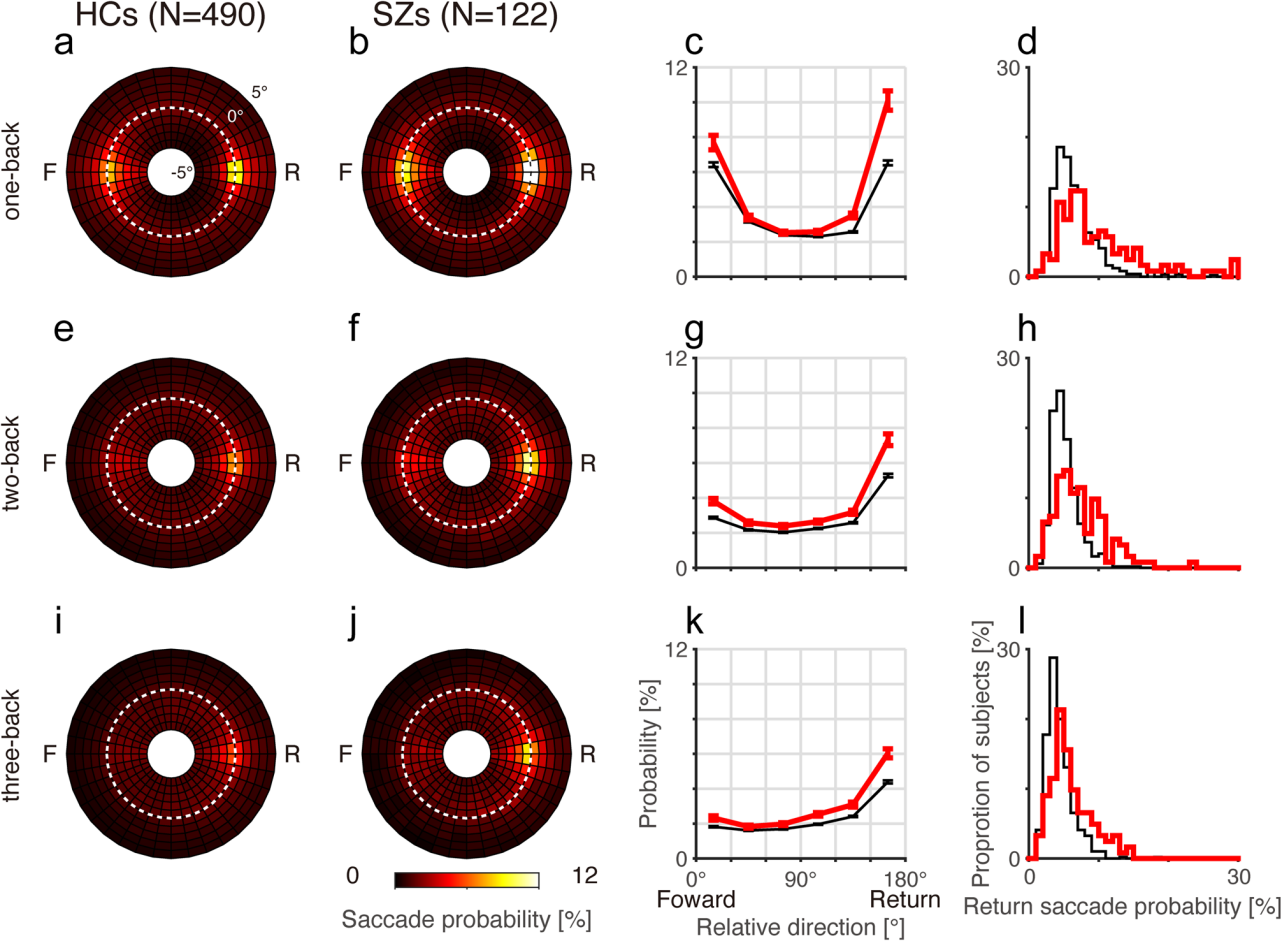
Mean saccade probability maps for the one-back (top), two-back (middle), and three-back (bottom) fixation locations for HCs (**a**,**e**,**i**) and SZs (**b**,**f**,**j**). Conventions are the same as in Fig. 2a,b. (**c**,**g**,**k**) Mean saccade probabilities plotted against relative saccade directions for amplitude-matched saccades for HCs (thin black lines) and SZs (thick red lines). Error bars indicate standard errors. (**d**,**h**,**l**) Frequency histograms of the return saccade probability for HCs (thin black lines) and SZs (thick red lines).

SZs also demonstrated an elevated frequency of return saccades compared to HCs. For the one-back fixation locations, return saccades were frequent in HCs (Fig. 3a) but even more common in SZs (Fig. 3b–d). The Friedman test revealed that saccade probability differed depending on the relative saccade direction in both HCs (*χ*^2^ = 1624, *p* < 0.001) and SZs (*χ*^2^ = 357, *p* < 0.001). Post-hoc analysis revealed that in both HCs and SZs, saccade probabilities were higher in return and forward directions than in the other four orthogonal directions (Fig. 3c, Wilcoxon signed-rank test, *p* < 0.001 after Bonferroni correction). When comparing the SZs and HCs, the effect size was largest for the probability of return directed saccades (Fig. 3d, 10.1 ± 6.1% for SZs, 6.5 ± 3.1% for HCs; *z* = 6.80, *p* < 0.001, *d’* = 0.92, AUC = 0.70)

.

Impairment of IOR in SZs persisted over a few cycles of fixations and saccades (Fig. 3e–l). SZs made more return saccades than HCs to two-back (7.3 ± 3.7% for SZs, 5.3 ± 2.2% for HCs; *z* = 6.06, *p* < 0.001, *d’* = 0.80, AUC = 0.68) and three-back locations (6.0 ± 2.9% for SZs, 4.4 ± 1.9% for HCs; *z* = 6.35, *p* < 0.001, *d’* = 0.76, AUC = 0.69).

The saccade amplitude was smaller in SZs than in HCs, leading to more amplitude-matched saccades in SZs (29.8 ± 9.3%) than in HCs (23.4 ± 5.7%; *z* = 7.68, *p* < 0.001). To determine whether the increase in return saccades in SZs merely reflected a greater number of amplitude-matched saccades, we compared the occurrence ratio of return saccades to that of amplitude-matched saccades. We found that even in this analysis, SZs made more return saccades than HCs (32.8 ± 12.6% for SZs, 27.6 ± 9.2% for HCs; *z* = 4.21, *p* < 0.001, *d’* = 0.52, AUC = 0.62).

To examine the effect of image salience on IOR, we analysed the return saccade probability when the participants viewed noise images. There was no difference in the salience of fixated locations between SZs and HCs (two-way ANOVA, F_(1,1167)_ = 2.04, *p* = 0.15) or between return and no-return saccade locations (F_(1,1167)_ = 0.75, *p* = 0.39), suggesting that the weak fluctuation of salience in the noise images had little if any effect on determining the next saccade location. Nevertheless, SZs made more frequent return saccades than HCs when viewing noise images (20.5 ± 22.1% for SZs, 9.2 ± 10.1% for HCs; Mann–Whitney *U*-test, *z* = 5.08, *p* < 0.001, *d’* = 0.85, AUC = 0.65). The result indicates that the frequent return saccades in SZs involve additional deficits other than a deficit in salience computation.

**Figure 4:**
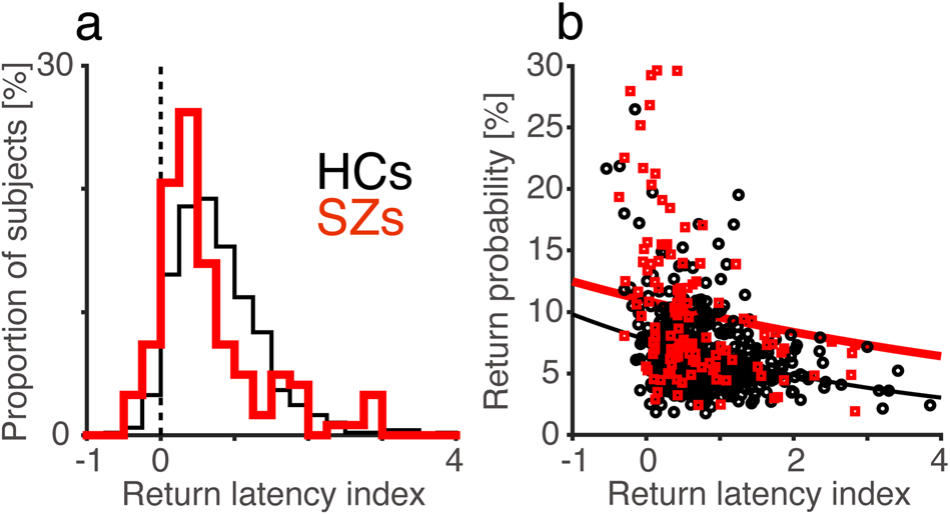
Relationship between return saccade probability and return latency index. (**a**) Frequency histograms of the return latency index for HCs (N = 490, thin black line) and SZs (N = 122, thick red line). (**b**) The relationship between the return saccade probability and return latency index for HCs and SZs. GLM analysis showed significant effects of the return latency index (coefficient = − 0.23 ± 0.03, *t* = − 6.8, *p* < 0.001) and group (coefficient = 0.34 ± 0.06, *t* = 5.4, *p* < 0.001), but not of the interaction (coefficient = 0.10 ± 0.06, *t* = 1.7, *p* = 0.09). Thin black and thick red lines represent regression lines estimated by the GLM model for HCs and SZs, respectively.

The effect of previously fixated locations on saccade latency was also compromised in SZs. The return latency indices of both SZs (0.68 ± 0.87, N = 122, Fig. 4a) and HCs (0.79 ± 0.61, N = 490) were significantly larger than 0 (sign test, *p* < 0.001), indicating that the latencies were longer for return saccades than for forward saccades. The return latency index was smaller in SZs than in HCs (Mann–Whitney *U*-test, *z* = 3.66, *p* < 0.001, *d’* = 0.16, AUC = 0.61), showing a weakened effect of the previous fixation location on the latency of the next saccade in SZs.

The probability and latency of return saccades were related to each other. For the one-back condition, GLM analysis showed that both HCs and SZs with a higher return saccade probability had a smaller return latency index (Fig. 4b; coefficient = − 0.23 ± 0.03, *t* = − 6.8, *p* < 0.001). The analysis also showed that the return saccade probability was higher in SZs than in HCs (coefficient = 0.34 ± 0.06, *t* = 5.4, *p* < 0.001). There was no interaction between return latency index and participant group (coefficient = 0.10 ± 0.06, *t* = 1.7, *p* = 0.09). Similar results were obtained for the two-back and three-back locations (Supplementary Note). These results suggest that the frequent return saccades and smaller return latency index were two phenotypes resulting from the compromised IOR in SZs.

### Attenuated IOR in SZs is causally associated with restriction of visual exploration

**Figure 5:**
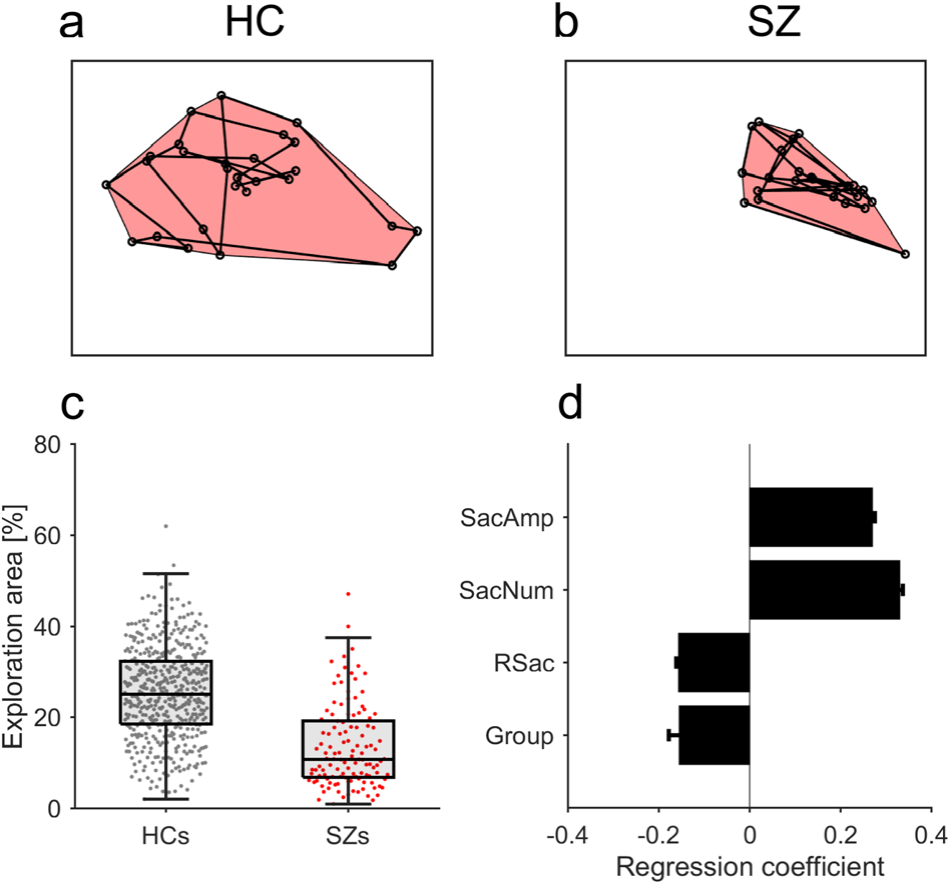
Contribution of return saccades to efficient visual search. (**a**,**b**) Regions explored by a representative HC (**a**) and SZ (**b**) are enclosed by connecting the outermost fixation locations (red shaded areas). The exploration area is defined as the proportion of the area enclosed by the outermost fixation locations relative to the entire image. (**c**) Box and beeswarm plots for the mean exploration area across images for HCs (N = 490) and SZs (N = 122). (**d**) Regression results of the GLME model to explain the exploration area based on saccade parameters. *SacAmp* saccade amplitude, *SacNum* total saccade number, *RSac* return saccade probability, *Group* HCs/SZs. Error bars indicate standard errors. All four parameters show significant effects (*p* < 0.001).

SZs searched smaller areas in visual images than HCs. For example, the exploration area of the HC shown in Fig. 2a was 31.3% (Fig. 5a), while that of the SZ shown in Fig. 2b was 8.4% (Fig. 5b). Population analysis revealed a marked difference in exploration area between SZs and HCs (Fig. 5c); SZs scanned only half the area of HCs (mean ± s.d., 13.6 ± 9.3% for SZs, 25.4 ± 10.1% for HCs; Mann–Whitney *U*-test, *z* = 10.61, *p* < 0.001, *d’* = 1.19, AUC = 0.81). Even when we calculated the exploration area separately for three equally divided periods of an entire test session, SZs explored smaller areas than HCs in every period including the first 3-min period (Mann–Whitney *U*-test, *p* < 0.001 after Bonferroni correction for all three comparison).

We next investigated the extent to which SZs’ frequent return saccades reduced the efficiency of their visual search. This efficiency may also have been impacted by the fact that the amplitude and number of saccades were smaller in SZs than in HCs. We therefore constructed a GLME model to estimate how the saccade amplitude (SacAmp), saccade number (SacNum), return saccade probability (RSac), and participant group (Group) contributed to the exploration area in each image category (Fig. 5d). The model analysis indicated that (i) exploration area increased with an increase in both the mean saccade amplitude (coefficient = 0.330 ± 0.007, *t* = 50.6, *p* < 0.001) and the number of saccades (coefficient = 0.269 ± 0.007, *t* = 40.6, *p* < 0.001), (ii) exploration area decreased with an increase in the return saccade probability (coefficient = − 0.156 ± 0.005, *t* = − 34.4, *p* < 0.001), and (iii) SZs showed a smaller exploration area than HCs (coefficient = − 0.154 ± 0.024, *t* = − 6.4, *p* < 0.001). Importantly, this full model explained the exploration area better than a reduced model that did not incorporate return saccade probability (*p* < 0.001, likelihood ratio test, AIC of full model = 342 and reduced model = 1198, adjusted *R*^2^ of full model = 0.93 and reduced model = 0.89). The fit of another reduced model that did not take participant groups into account was slightly worse than that of the full model, but both had similar *R*^2^ values (*p* < 0.001, likelihood ratio test, AIC of reduced model = 379, adjusted *R*^2^ = 0.93). These results suggest that a higher probability of return saccades, smaller saccade amplitude, and lower number of saccades almost fully explain the restricted scanning in SZs.

### Correlations between IOR impairment and clinical characteristics

**Figure 6:**
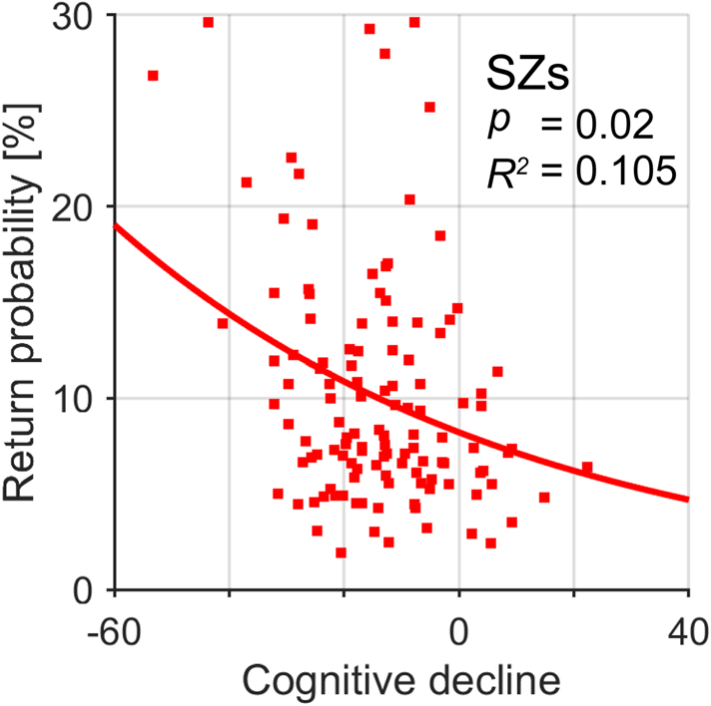
Relationship between return saccade probability and cognitive decline for SZs. Line represents the regression line estimated by the GLM model. Bonferroni-corrected *p* values and adjusted *R*^2^ are shown.

The relationships between SZs’ return saccade probability and their demographic and clinical characteristics were determined by constructing separate GLMs for HCs and SZs (Table 2). In SZs, IOR impairment was significantly correlated only with cognitive decline (difference between premorbid IQ and current IQ); the return saccade probability was higher in subjects with a large cognitive decline (Fig. 6; RSac = 8.2 × exp[− 0.014 × Cognitive decline]; *p* = 0.02 after Bonferroni correction, adjusted *R*^2^ = 0.105). Return saccade probability in SZs was not significantly correlated with the duration of illness, PANSS scores, or dose of antipsychotics (Table 2). In HCs, there were weak but statistically significant relationships between some demographics with return saccade probability and exploration area, but the *R*^2^ of the model was very small (Table 2).

**Table 2:** Regression coefficients for return saccade probability and exploration area explained by the demographic and clinical characteristics of HCs and SZs.

	Return saccade probability			Exploration area		
	Coefficient	Adjusted *R*^2^	*p* value	Coefficient	Adjusted *R*^2^	*p* value
**Healthy controls (N = 490)**						
Demographics						
Age (years)	0.003	0.009	0.07	**− 0.005**	**0.029**	**< 0.001**
Education (years)	**− **0.011	0.000	0.99	0.023	0.008	0.41
Current IQ	**− 0.006**	**0.029**	**0.001**	**0.007**	**0.039**	**< 0.001**
**Schizophrenia (N = 122)**						
Demographics						
Age (years)	**− **0.001	**− **0.008	1.00	**− **0.008	0.009	1.00
Education (years)	0.000	**− **0.008	1.00	**− **0.015	**− **0.005	1.00
Current IQ*	**− **0.009	0.033	0.21	0.008	0.026	0.49
Estimated premorbid IQ*	0.004	**− **0.005	1.00	0.001	**− **0.008	1.00
Cognitive decline*	**− 0.014**	**0.105**	**0.02**	0.015	0.041	0.07
Onset age (years)	0.000	**− **0.008	1.00	0.000	**− **0.008	1.00
Duration of illness (years)	**− **0.001	**− **0.008	1.00	**− **0.001	**− **0.008	1.00
Symptoms						
PANSS total	0.003	**− **0.003	1.00	0.003	**− **0.003	1.00
PANSS positive	**− **0.001	**− **0.008	1.00	**− **0.001	**− **0.008	1.00
PANSS negative	0.021	0.008	0.78	0.021	0.008	0.78
PANSS general	0.007	**− **0.002	1.00	0.007	**− **0.002	1.00
Drug (mg/day)						
CPZ eq.	0.000	**− **0.008	1.00	0.000	**− **0.008	1.00
Typical antipsychotic eq.	0.000	0.007	1.00	0.000	0.007	1.00
Atypical antipsychotic eq.*	0.000	0.004	1.00	0.000	0.004	1.00
Diazepam eq.*	**− **0.003	**− **0.005	1.00	**− **0.003	**− **0.005	1.00
Biperiden eq.*	**− **0.118	0.044	0.45	**− **0.118	0.044	0.45

Bonferroni-corrected *p* values (raw *p* value × 3 for HCs, × 16 for SZs) and adjusted *R*^2^ are shown. A negative adjusted R^2^ is interpreted as zero variance explained. Bold underlined values highlight statistically significant contributions (*p* < 0.05).

*In SZs, N = 121 for current IQ, estimated premorbid IQ, cognitive decline, and atypical antipsychotic eq., N = 94 for diazepam eq., and N = 80 for biperiden eq.

*PANSS* Positive and Negative Syndrome Scale, *CPZ* *eq*. chlorpromazine equivalents.

SZs had a lower current IQ than HCs (Table 1). To check whether the difference in current IQ could explain the difference in the return saccade probability between SZs and HCs, we resampled 74 participants from each group who were matched regarding current IQ (97 ± 9 for SZs, 98 ± 9 for HCs; Mann–Whitney *U*-test, *z* = 0.82, *p* = 0.41). Even in these IQ-matched samples, SZs exhibited more return saccades than HCs (10.2 ± 7.1 for SZs, 7.7 ± 5.1 for HCs; *z* = 3.08, *p* = 0.002). Thus, a lower current IQ is not likely to be a major factor accounting for the higher return saccade probability in SZs.

## Discussion

In the present investigation we studied the properties of exploratory saccades in SZs and compared them with those in HCs. We found that (i) SZs had impairments in IOR under free-viewing conditions, (ii) SZs explored a smaller area of the visual scene, (iii) the attenuated IOR and the resulting repeated visits to previously explored regions were a partial cause of the limited scanning, and (iv) the frequency of return saccades in SZs was related to cognitive decline (i.e., the difference between premorbid IQ and current IQ) but not to the dose of prescribed antipsychotics.

In free-viewing conditions, the current sample of SZs showed a smaller saccade amplitude, smaller saccade number, and longer duration of fixation, compared to those of HCs, which is consistent with previous reports^6,19–22^. Impairments of IOR in SZs manifested as frequent return saccades and as a smaller difference in the latency between return and forward saccades (Figs. 2, 3, 4). Thus, the IOR was compromised both spatially and temporally in SZs. These findings extend the IOR impairment in SZs from Posner’s paradigm^32–34^ to a more naturalistic, free-viewing setting. The frequent return saccades in this setting may result from the aberrant computation of salience of visual scenes in SZs^16^. However, SZs returned their fixation to previously fixated locations more frequently than HCs even when they viewed 1/f noise stimuli with a weak change in salience across space. This finding indicates that problems with salience computation do not explain all aspects of the frequent return saccades in SZs. A deficit in endogenous, top-down control of attention processes, which is thought to underly the IOR impairment in Posner’s paradigm for SZs, could also cause the IOR impairment in free-viewing condition^33^. The deficit of attention in which SZs allocate their processing resources to a small number of representations and fail to distribute attention among multiple locations^50^ may well explain the IOR impairment.

According to the foraging facilitator hypothesis, impaired IOR in SZs results in an inefficient search of visual scenes. Many previous studies reported that visual exploration was confined to a narrow region in SZs^1–4^, but the evidence was descriptive. Only a few studies provided quantitative evidence such as more clustered fixations in SZs compared to HCs^20,21^. We found a striking reduction in the extent of visual exploration in SZs, and using GLME model analysis, we further demonstrated that impaired IOR in SZs led to restricted visual exploration (Fig. 5). The clustered fixations in SZs could be partly caused by impaired IOR and the resultant inappropriate and repetitive viewing.

The repeated visits to previous fixation locations and limited visual scene exploration are unlikely to be due to the motivational deficits that comprise one component of the negative symptoms in SZs, because we found no relationship between PANSS scores and either return saccade probability or exploration area (Table 2). Furthermore, the restricted visual exploration of SZs was observed even when we confined our analysis to the first third of an experimental session, suggesting that there would be mechanisms other than deficits in keeping attention and motivation. However, we did not consider the detailed aspects of cognitive dysfunction in individual SZs, such as problems with selective attention, sustained attention, motivation, and visual processing^18^. The relationship between IOR impairment and detailed cognitive (dis)ability needs to be clarified in future studies.

The impaired IOR in SZs could impede efficient visual search and appropriate information gathering from visual scenes and thus influence daily behaviour. Previous studies found that eye movement abnormalities in SZs, such as smaller and fewer saccades, were associated with atypical performance in a number of cognitive tests that require appropriate information gathering^12–15^. For example, to properly perform the Benton Visual Retention test, participants must detect and memorize fine features of an image. However, SZs often look at only a few stereotypical spots within the image^14^, which is very similar to the visual search behaviour of our SZs. The atypical performance on cognitive tests by SZs may at least partially reflect impaired IOR and the resultant inappropriate information gathering.

Greater cognitive decline was associated with more impaired IOR in SZs (Fig. 6). A lower current IQ in SZs did not explain the higher return saccade probability, because SZs showed more return saccades than IQ-matched HCs. The attenuation of IOR in SZs thus may reflect the degree of disease-related cognitive degradation, but not the current IQ. Previous studies in HCs revealed that working memory plays a role in IOR^51,52^. Although working memory capacity may be partially captured by current IQ, further studies are needed to gain a better understanding of the relationship between working memory and IOR impairment in SZs.

Schizophrenia has been associated with hyperactivity of the striatal dopamine D2 receptors as well as elevated striatal dopamine synthesis and release capacity. The glutamatergic system has also been proposed to contribute to the pathophysiology of SZs via hypofunction of glutamatergic transmission through NMDA receptors in cortical areas and increased glutamate levels, although the evidence is inconsistent^53^. In this study, the return saccade probability was not related to the dosage of typical or atypical antipsychotics that target dopaminergic D2 receptors (Table 2). Several previous studies reported possible relationships between various neurotransmitters and IOR impairment in SZs. Short-term administration of a dopamine D2 receptor antagonist was not related to IOR in SZs under Posner’s paradigm^54^. On the other hand, administration of the NMDA antagonist ketamine to HCs, which is a potential model of schizophrenia, blunted the IOR in Posner’s paradigm^55,56^. Future studies will clarify the roles of specific neurotransmitters in IOR impairment in SZs.

Eye movement abnormalities have been proposed as a biomarker for SZs^5–8^. However, previously reported eye movement abnormalities, such as difficulty in executing voluntary saccades and poor performance in smooth pursuit, were also related to patients’ drug treatment histories, raising the possibility that these abnormalities simply reflect side effects of pharmacological treatment on the oculomotor system^6,57–60^. By contrast, the IOR impairment we investigated in this study was not related to dopaminergic medication effects or duration of illness (Table 2). We do not have data about treatment duration, which is another possible factor affecting the brain in SZs^61^. Although IOR impairment was not ubiquitous among SZs, it may serve as a useful and auxiliary marker in this population.

Eye movement analysis is expected to be an affordable and feasible tool to assess pathophysiology in psychiatric disorders, changes in cognitive ability with development and aging, and mental conditions such as fatigue, sleepiness, and stress. IOR examination under free-viewing conditions may be applied to other clinical and non-clinical populations (e.g., the first-degree relatives of SZs). A technical advantage of the paradigm we used is its feasibility; the test does not require that subjects receive any specific prior training, and it is even applicable to infants. Furthermore, the availability of eye trackers using webcams, smartphones, and smart glasses will create new opportunities for examining eye movements and IOR in daily life.

## Supplementary Information

Supplementary Information.

## Data Availability

The datasets generated and/or analysed during the current study are not publicly available because they contain information that could compromise research participant privacy and consent, but are available from the corresponding author on reasonable request.
