# Supplementary material for: Impaired inhibition of return during free-viewing behaviour in patients with schizophrenia
Source: Sci Rep. 2021 Feb 5;11:3237. doi: 10.1038/s41598-021-82253-w (PMC7865073; doi:10.1038/s41598-021-82253-w)
Supplement: Supplementary file 1 — Supplementary Information. [file 41598_2021_82253_MOESM1_ESM.docx]

**Supplementary Information**

**Impaired inhibition of return during free-viewing behaviour**

**in patients with schizophrenia**

Ken-ichi Okada, Kenichiro Miura, Michiko Fujimoto, Kentaro Morita, Masatoshi Yoshida, Hidenaga Yamamori, Yuka Yasuda, Masao Iwase, Mikio Inagaki, Takashi Shinozaki, Ichiro Fujita, Ryota Hashimoto

**Supplementary Table S1, Summary of IOR in study participants.**

| HCs | Higher probability  of return saccades | Equal or lower probability of return saccades | Total |
| --- | --- | --- | --- |
| Delayed latency  of return saccades | 71 (15%) | 290 (59%) | 361 (74%) |
| Less-delayed latency of return saccades | 55 (11%) | 74 (15%) | 129 (26%) |
| Total | 126 (26%) | 364 (74%) | 490 |

| SZs | Higher probability  of return saccades | Equal or lower probability of return saccades | Total |
| --- | --- | --- | --- |
| Delayed latency  of return saccades | 18 (15%) | 52 (42%) | 70 (57%) |
| Less-delayed latency of return saccades | 34 (28%) | 18 (15%) | 52 (43%) |
| Total | 52 (43%) | 70 (57%) | 122 |

The proportion of participants who exhibited strong IOR according to both equal or lower probability of return saccades and delayed latency of return saccades was smaller in SZs (52/122, 42%) than in HCs (290/490, 59%; *χ^2^*-test, χ^2^ = 10.9, *p* < 0.001). In other words, the remaining 58% of SZs showed attenuated IOR according to a higher probability of return saccades, less-delayed latency of return saccades, or both.

**Supplementary Note, Effects of the two-back and three-back fixation locations on saccade latency**

We analyzed the effects of two- and three-back fixation locations on saccade latency. Because we had few return and forward saccades relative to two- and three-back fixation locations, it was difficult to obtain sufficiently stable baseline data from forward saccades to calculate the return latency index. We used the latency for saccades with a wider variety of directions (amplitude difference < 1°; direction difference < 90°) as a control to calculate the return latency index.

We first confirmed that this new return latency calculation yielded similar results for those calculated by using the latency for forward saccades described in the main text. The return latency indices for one-back locations of both SZs (mean ± s.d., 0.48 ± 0.49) and HCs (0.62 ± 0.47) were significantly larger than 0 (sign test, *p* < 0.001), indicating that the latencies were longer for return saccades than for saccades with amplitude difference < 1° and direction difference < 90°. The return latency index was smaller in SZs than in HCs (Mann–Whitney *U*-test, *z* = 3.38, *p* < 0.001, *d’* = 0.28, AUC = 0.60), showing a weakened effect on the saccade latency in SZs. GLM analysis showed that the temporal and spatial aspects of IOR were related to each other. The return probability was higher for SZs and HCs with a smaller latency index (coefficient = −0.31 ± 0.04, *t* = −7.29, *p* < 0.001), and it was higher in SZs than in HCs (coefficient = 0.49 ± 0.07, *t* = 7.54, *p* < 0.001). There was a weak interaction between latency and subject group (coefficient = −0.26 ± 0.09, *t* = −2.83, *p* = 0.005).

We then analyzed the effects of two- and three-back fixation locations on saccade latency. The return latency indices for two-back locations (0.37 ± 0.47 for SZs and 0.34 ± 0.42 for HCs; *p* < 0.001) and three-back locations (0.27 ± 0.64 for SZs and 0.22 ± 0.45 for HCs; *p* < 0.001) were significantly larger than 0, indicating that return saccades to locations of two- or three-back fixations were slowed compared to forward saccades for both SZs and HCs. There were no significant differences between SZs and HCs in the return latency index for two-back (*z* = 0.10, *p* = 0.92, *d'* = 0.07, AUC = 0.50) or three-back (*z* = 0.21, *p* = 0.83, *d'* = 0.10, AUC = 0.49) locations. GLM analysis showed that the return probability was higher for subjects with smaller latency modulation, and the return probability was higher in SZs than in HCs, both for two-back locations (coefficient for return latency index = −0.19 ± 0.05, *t* = −4.11, *p* < 0.001, coefficient for group = 0.36 ± 0.06, *t* = 6.59, *p* < 0.001, coefficient for interaction = -0.10 ± 0.09, *t* = -1.02, *p* = 0.31) and three-back locations (coefficient for return latency index = −0.15 ± 0.04, *t* = −3.43, *p* < 0.001, coefficient for group = 0.31 ± 0.05, *t* = 6.32, *p* < 0.001, coefficient for interaction = 0.06 ± 0.08, *t* = 0.83, *p* = 0.41).
